# Involvement of microglial P2X7 receptor in pain modulation

**DOI:** 10.1111/cns.14496

**Published:** 2023-11-10

**Authors:** Jing Zhang, Lei Gao, Yaoyuan Zhang, Haozhen Wang, Shukai Sun, Li‐an Wu

**Affiliations:** ^1^ State Key Laboratory of Oral & Maxillofacial Reconstruction and Regeneration, National Clinical Research Center for Oral Diseases, Department of Pediatric Dentistry, School of Stomatology The Fourth Military Medical University Xi'an Shaanxi China

**Keywords:** microglia, P2X7R, pain

## Abstract

**Background:**

Pain is a rapid response mechanism that compels organisms to retreat from the harmful stimuli and triggers a repair response. Nonetheless, when pain persists for extended periods, it can lead to adverse changes into in the individual’s brain, negatively impacting their emotional state and overall quality of life. Microglia, the resident immune cells in the central nervous system (CNS), play a pivotal role in regulating a variety of pain‐related disorders. Specifically, recent studies have shed light on the central role that microglial purinergic ligand‐gated ion channel 7 receptor (P2X7R) plays in regulating pain. In this respect, the P2X7R on microglial membranes represents a potential therapeutic target.

**Aims:**

To expound on the intricate link between microglial P2X7R and pain, offering insights into potential avenues for future research.

**Methods:**

We reviewed 140 literature and summarized the important role of microglial P2X7R in regulating pain, including the structure and function of P2X7R, the relationship between P2X7R and microglial polarization, P2X7R‐related signaling pathways, and the effects of P2X7R antagonists on pain regulation.

**Results:**

P2X7R activation is related to M1 polarization of microglia, while suppressing P2X7R can transfer microglia from M1 into M2 phenotype. And targeting the P2X7R‐mediated signaling pathways helps to explore new therapy for pain alleviation. P2X7R antagonists also hold potential for translational and clinical applications in pain management.

**Conclusions:**

Microglial P2X7R holds promise as a potential novel pharmacological target for clinical treatments due to its distinctive structure, function, and the development of antagonists.

## Graphical abstract

In this review, we first generalized the relationship between microglial P2X7R and polarization in pain modulation. In addition, the P2X7R‐mediated pain signaling pathways were summarized. Finally, we discussed the application and progress of P2X7R antagonists in pain therapy.

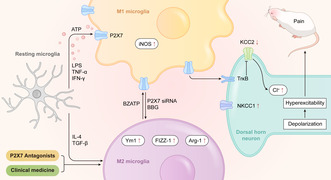

## 1 INTRODUCTION

According to the International Association for the Study of Pain (IASP), pain is defined as an unpleasant sensation linked to actual or potential tissue damage, disrupting emotional cognition and affecting physiological responses.^1^ Healthy individuals may experience brief moments of pain, with their neural mechanisms held in an inactive state, which is ready to respond when the body is under threat. Due to pain, people may even suffer from a series of complications. While pharmacological therapy including non‐steroidal anti‐inflammatory drugs and opioids^2^ remains a prevalent approach to pain management, sustained analgesic effects are challenging to achieve, and the clinical side effects of drugs pose issues for medical practitioners. In addition, physical stimulation and traditional Chinese medicine therapy have demonstrated efficacy in pain relief and modulation.^3^, ^4^ Consequently, effective pain management remains a significant challenge, which requires more attention and further research for resolution.

The concept of purinergic signaling was initially introduced in 1972^5^ and followed by the definition of purinergic receptors in 1976.^6^ Receptors for purines are extensively involved in immune responses, inflammation and pain pathways.^7^ Subsequently, the first nucleotide receptors, termed P2 purinoceptors, were cloned in 1993.^8^, ^9^ Among these, the P2X receptors belonging to the category of ligand‐gated ion channels encompass seven subtypes, ranging from P2X1R to P2X7R. P2X7R is recognized as the last subtype and also referred to as P2Z receptor,^10^ which holds implications in a wide spectrum of diseases.^11^, ^12^, ^13^ Especially, it is predominant in microglia and assumes a pivotal role in nociceptive sensitivity.^14^, ^15^, ^16^

Microglia are not only neuroglial cells but also function as immune cells that sustain the equilibrium between physiological and pathological states to uphold brain health.^17^ And disruption of brain homeostasis including injury and inflammation will lead to their activation.^18^ It is widely acknowledged that microglia contribute to the emergence and persistence of pain.^19^ Notably, P2X7R holds significance within the microglia–neuron signaling system for pain regulation.^11^, ^20^ And there is an increasing consensus, suggesting that P2X7R is interconnected with various pain‐associated signaling pathways such as inflammasomes and transcription factors.^21^, ^22^, ^23^, ^24^ In recent years, many scholars have directed their attention to the interplay between microglial P2X7R and pain.^25^ This review summarizes recent advances in pain modulation, uncovering the pivotal role of microglial surface receptor P2X7 in the pain signaling process.

## 2 AN OVERVIEW OF P2X7R

### 2.1 The basic structure and properties of P2X7R

P2X7R was first discovered in 1998, pinpointing its location near human chromosome 12q24.^26^ It represents the seventh isoform of the P2X receptors and is unique among this protein family in that it is dependent on adenosine 5′‐triphosphate (ATP) non‐selective cation channel.^27^ Comprising three primary domains,^10^, ^28^, ^29^ P2X7R includes an extracellular loop, the ATP binding site, and two structural transmembrane domains (TM1 and TM2), accountable for regulating the ion channel's opening. Importantly, ATP concentrations can influence the activation of pre‐ and postsynaptic P2X receptors, consequently influencing pain signaling dynamics.^30^ The physiological dormancy of P2X7R prevails due to the controlled, low levels of ATP in a healthy state. As such, P2X7R is primarily activated at higher concentrations of the endogenous ligand ATP (>100 μM).^31^, ^32^ Upon activation, P2X7R will be regarded as a mediator of excitatory neurotransmission in both the central and peripheral nervous systems.^33^ Meanwhile, P2X7R is involved in inflammatory processes associated with the intracellular communication between neurons and glia.^15^, ^34^, ^35^ For instance, microglia secreted exosomes in response to the stimulation of P2X7R, and the inflammatory signaling contained in exosomes is transferred into neurons.^36^ Additionally, activation of microglial P2X7R triggers the translocation of P2X4 receptors to microglial surfaces and releases of interleukin‐1β (IL‐1β) and brain‐derived neurotrophic factor (BDNF).^37^ These factors impact neurons, influencing their excitability states and instigating pain.

### 2.2 Diverse localization of P2X7R

Albeit P2X7R is primarily localized within microglia in the CNS, it has been documented in satellite glial cells, astrocytes, and some neurons. Importantly, it is intricately linked to the pain response.^38^, ^39^, ^40^, ^41^ In satellite glial cells, P2X7R functions in the extracellular signal‐regulated kinase 1 (ERK) signaling pathway to mediate the pain response and is modulated by the transient receptor potential vanilloid receptors 1 and 4 (TRPV1 and TRPV4).^42^, ^43^, ^44^ Wu et al.^45^ recently established a chronic compression injury model and uncovered a novel transcriptional mechanism where BDNF prompts P2X7R expression in dorsal root ganglion neurons. Besides its presence in brain cells, P2X7R is also expressed in the salivary gland epithelium, with heightened expressed in patients afflicted with primary desiccation syndrome.^46^ Furthermore, P2X7R expression is upregulated in the pulpitis tissue in human and rats.^47^, ^48^ On all accounts, P2X7R is a pivotal molecular expressed throughout the body and primarily concentrated on the microglia.

## 3 P2X7R REGULATES THE ACTIVATION AND POLARIZATION STATES OF MICROGLIA

Microglia located in the CNS play an important role in pain regulation due to their own functions.^49^ And P2X7R‐mediated activation and polarization of glia are often closely related to the occurrence, development, and regulation of inflammation. In addition, the states of activation and polarization influence the inflammatory process.^50^ Therefore, we discussed the role of P2X7R in the activation and polarization of microglia.

### 3.1 P2X7R drives microglial activation

P2X7R plays a pivotal role in microglial activation,^51^ where its overexpression can heighten microglial membrane permeability, contribute to microgliosis, promote release of inflammatory factors, and further induce neuronal damage.^52^ Resting microglia possess small cytosomes with elongated branches, whereas activated microglia manifest larger cytosomes with shorter, robust extensions resembling amoebas. Specific surface markers such as Mac‐1, Iba‐1, CD11b, and OX42 have been documented on activated microglia,^53^, ^54^, ^55^ highlighting their distinctive features. The activation of microglia requires the involvement and regulation of calcium ions, such that elevated levels of ATP stimulate an influx of Ca^2+^ into microglia that lead to the opening of P2X7 receptor ion channels and the activation of microglia.^26^, ^56^, ^57^ Apart from ATP, microglia can also be activated by lipopolysaccharides (LPS) and P2X7R agonists in vitro,^58^ warranting further investigation into the morphological, molecular, and electrophysiological aspects of P2X7R function and microglial activation.

### 3.2 P2X7R regulates microglial polarization

**FIGURE 1:**
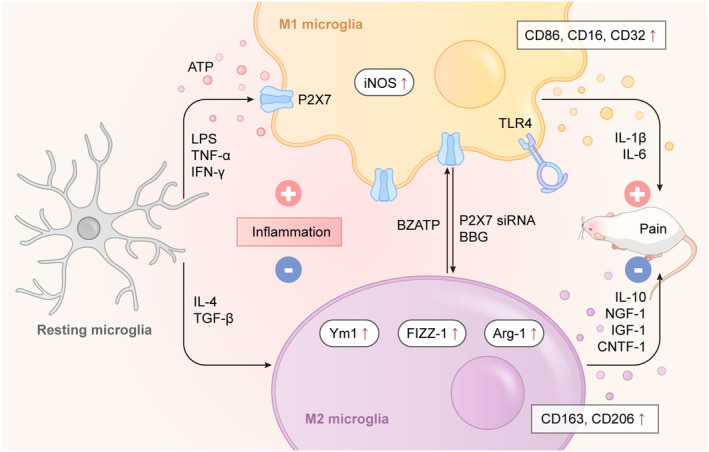
The relationships between microglial phenotypes and P2X7R in pain modulation. Microglia can acquire two different phenotypes that express different surface markers and receptors under different condition. LPS, TNF‐α, and IFN‐γ can turn microglia into M1 phenotype and induce painful feelings, in which they secrete inflammatory cytokines such as IL‐1β and IL‐6. In contrast, administration of IL‐4 and TGF‐β can induce microglia M2 phenotype and release anti‐inflammatory factors such as IL‐10 and IGF‐1. Notably, P2X7R activation is related to M1 microglia, while suppressing P2X7R can transfer M1 microglia into M2 phenotype. BBG or P2X7R siRNA promotes M2 phenotype and alleviates pain via inhibiting P2X7R‐mediated inflammation, whereas BzATP has an opposite effect. And the balance between pro‐ and anti‐inflammatory mediators released from M1/M2 microglia is closely associated with P2X7R‐induced pain state.

In response to injury and inflammatory stimuli, microglia can undergo polarization into two distinct phenotypes: M1 pro‐inflammatory and M2 anti‐inflammatory states.^59^, ^60^, ^61^ These polarized microglia release disparate signaling molecules that exert distinct effects on target neurons to regulate pain. M1‐polarized microglia promote inflammatory responses and exert cytotoxic effects, while M2‐polarized microglia suppress inflammatory responses and exert tissue‐protective effects.^62^ The polarization of microglia to M1 phenotype is triggered by inducers such as tumor necrosis factor‐α (TNF‐α), interferon‐gamma (IFN‐γ), and LPS, whereas M2 polarization is induced by interleukin‐4 (IL‐4) and transforming growth factor‐β (TGF‐β).^63^, ^64^, ^65^, ^66^ M1 microglia express surface markers like CD86, CD16, and CD32,^61^ along with increased expression of inducible NOS (iNOS) and Toll‐like receptor 4 (TLR4), subsequently releasing pro‐inflammatory factors such as IL‐1β and IL‐6.^67^ On the contrary, M2 microglia specifically express CD163 and CD206, upregulate arginase‐1 (Arg1), Ym1 and FIZZ‐1, and secrete IL‐10, CNTF‐1, IGF‐1, and NGF‐1,^68^ which contribute to pain relief.^69^ Remarkably, modulating P2X7R expression can alter the direction and behavior of microglial polarization. Generally, microglial P2X7R is activated at high ATP levels, and then, the channel pore is open, allowing the passage of small cations. Upon activation, P2X7R can release neurotransmitters into the extracellular space in the CNS.^70^ Simultaneously, P2X7R activation is related to M1 microglia and is involved in the development and progression of inflammation. Additionally, studies also showed that P2X7R could serve as a potential molecular target for positron emission tomography (PET) imaging of M1 microglia.^71^, ^72^, ^73^ In the context of brain injury‐induced inflammation, P2X7R is linked to rat microglial M1/M2 polarization regulation.^74^ Moreover, in the chronic compression injury of the sciatic nerve (CCI) model, microglia were polarized to the M1 type and P2X7R expression was increased.^75^ Similarly, P2X7R mediated activated microglia toward the M1 phenotype in cancer‐induced bone pain. Notably, siRNA‐mediated P2X7R knockdown converted microglial polarization from the M1 type to M2 type and BBG had a similar effect, which significantly increased the expression of M2 markers and effectively alleviated pain (Figure 1).^30^ The above findings collectively suggest that P2X7R expression can influence the intensity and regression of pain by modulating microglial polarization.

## 4 MICROGLIAL P2X7R ASSOCIATED WITH VARIOUS TYPES OF PAIN

Acute pain has a high diagnostic value for tissue damage and facilitates healing by prompting protective responses like immobilization.^76^ However, when pain transitions to a chronic state, its diagnostic value diminishes, evolving into an independent condition that significantly impacts patient lives. Due to the diversity of functions and mechanisms of microglial P2X7R, as well as its active response in inflammation, P2X7R becomes a major focus of the pain field. With its diverse functions, mechanisms, and active role in inflammation, microglial P2X7R emerges as a central focus within the field of pain research. This section explores its contributions to different pain modalities, encompassing neuropathic, inflammatory, and cancer‐related pain.

### 4.1 The function of microglial P2X7R in neuropathic pain

Neuropathic pain, arising from somatosensory nervous system damage,^77^ has a prevalence ranging from 3% to 17%. It is related to inflammatory stimuli that elicit specific neuronal pathways generating nociception.^78^ In pathological states, microglial P2X7R is activated upon binding to ATP and is accompanied by increased release of inflammatory factors including TNF‐α, IL‐6, and IL‐1β.^79^ These cytokines are closely related to P2X7R‐mediated neuropathic pain. P2X7R is also responsible for tactile abnormalities and nociceptive hypersensitivity in models like the CCI, diabetic neuropathic pain (DNP), and spinal nerve ligation (SNL). In a CCI model, Xu et al.^80^ found that P2X7R agonist BzATP not only boosted microglial P2X7R expression but also potentiated the release of inflammatory factors including IL‐1β and IL‐18, and finally induced pain. Similarly, electroacupuncture treatment was found to mitigate P2X7R expression, alleviating neuroinflammation and pain.^27^, ^80^, ^81^ Last but not least, reduced pain sensitivity has been documented in P2X7R knockout mice,^56^, ^82^, ^83^ demonstrating the necessity of P2X7R in neuropathic pain.

**FIGURE 2:**
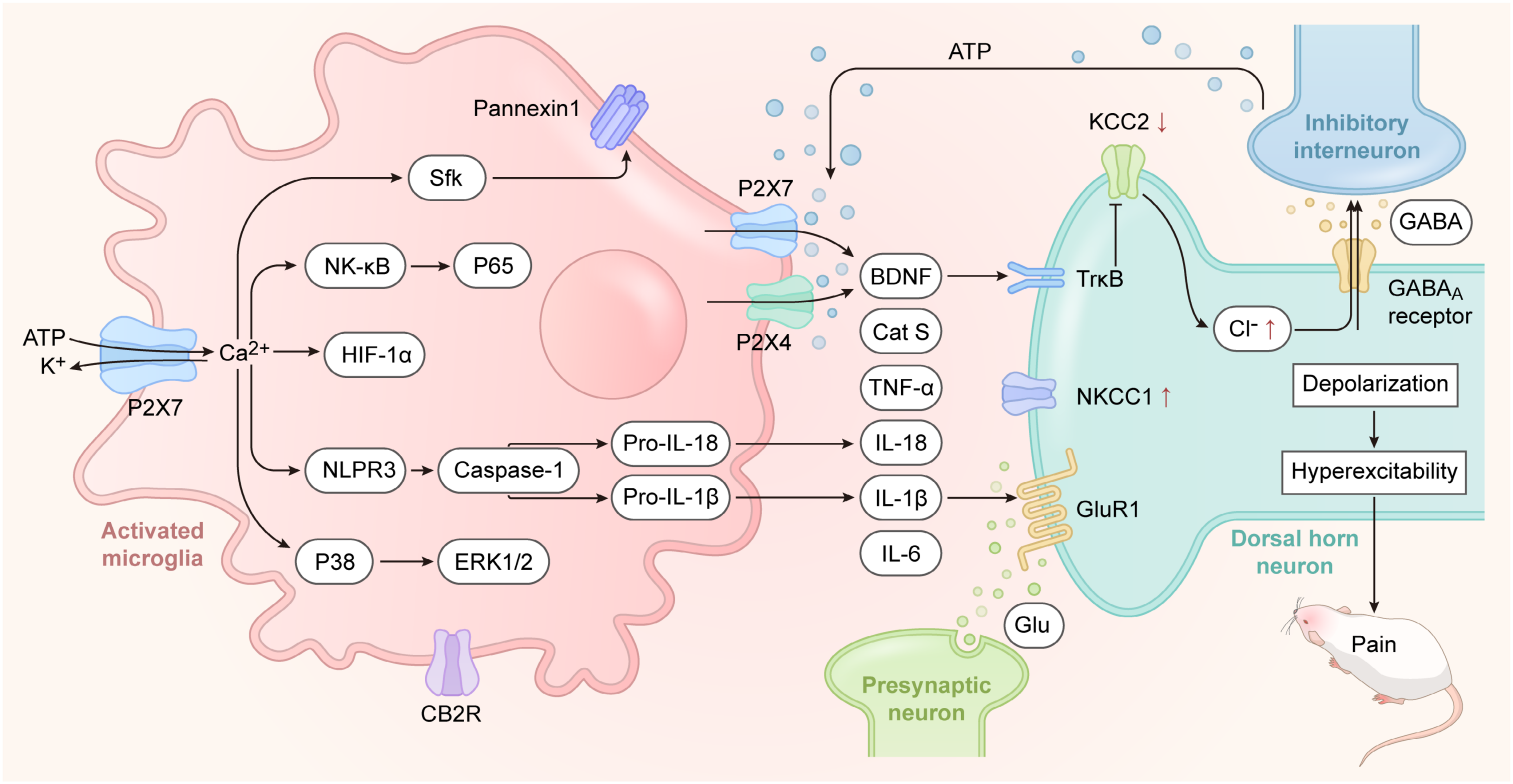
P2X7R‐mediated pain signaling pathways. P2X7R is activated when the concentration of ATP rises over 100 μM, opening the cell membrane‐bound ion channel and leading to an influx of calcium and efflux of potassium. As a consequence, several signaling pathways are activated, including the Src/pannexin1, NF‐κB/P65, P2X7R/HIF‐1α, NLRP3/caspase‐1, and P38/ERK1/2 pathways, together with the release of inflammatory factors, and IL‐1β causes an increase in GluR1. In addition, activation of microglial P2X7R makes migration of P2X4R to the microglial surface and promotes the release of BDNF. BDNF binds to TrkB receptor and downregulates the KCC2. KCC2 exports Cl^−^ out of the neurons, while NKCC1 promotes accumulation of Cl^−^ and together drives GABAergic depolarization. Accumulation and release of microglial inflammatory factors can evoke hyperexcitability potentials of neurons, finally leading to pain sensation.

Recently, more and more studies have explored the relevant molecular mechanisms linking microglial P2X7R with neuropathic pain. The P2X7R/p38 mitogen‐activated protein kinase (MAPK) cascade constitutes a classical signaling pathway associated with pain modulation. Activation of this pathway controls the release of the pro‐inflammatory cytokine IL‐1β from microglial microvesicles that directly stimulate neurons.^84^ Elevated release of IL‐1β causes an increase of GluR1 in postsynaptic neurons,^85^ ultimately inducing long‐term potentiation (LTP) in spinal cord injury responses involving microglial P2X7R and p38. Moreover, increased expression of P2X7R and p38 in medullary oblongata microglia triggered pain even in non‐injured skin.^86^ Activation of P2X7R through p38 MAPK also generates TNF‐α in microglia, fostering hyperalgesia.^87^ These findings provide the evidences of P2X7R in modulating pro‐inflammatory processing. Notably, persistent P2X7R activation contributes to the assembly of NLR family pyrin domain containing 3 (NLRP3) inflammasome, which can make pain even worse.^88^ For example, in a CCI model, P2X7R was shown to mediate the sustained release of inflammatory factors and promoted the formation of NLRP3 inflammatory vesicles, establishing a feedback loop sustaining abnormal pain.^89^ In addition, microglial P2X7R contributes to chronic pain maintenance by activating ATP‐permeable channel Pannexin 1 via src family kinase (sfk) signaling.^90^ And this process possibly was caused by the increase in the intracellular calcium.^91^ Cathepsin S is a lysosomal cysteine protease that released from microglia and regulated by P2X7R in neuropathic pain.^92^ P2X7R also promotes BDNF release, which can bind to TrkB receptor and compromise KCC2 function in the dorsal horn.^93^ Remarkably, the decreased expression of KCC2 was accompanied by upregulated Na^+^‐K^+^‐Cl^−^ co‐transporter 1 (NKCC1), promoting g‐aminobutyric acid (GABA) excitability. More importantly, KCC2 is responsible for transporting Cl^−^ ions out of the neurons, while NKCC1 promotes Cl^−^ accumulation, contributing to GABAergic depolarization.^94^ Thus, regulating BDNF–TrkB‐KCC2 pathway holds promise for pain management (Figure 2). Recent studies have shown that microglial P2X7R activation in chronic migraine states causes impaired autophagic flux, leading to the upregulation of autophagy‐related structural proteins that contribute to central sensitization in the trigeminal nerve's caudal nucleus.^95^ And autophagy also suppresses the expressions of pro‐inflammatory cytokines, further modulating pain behavior.^96^, ^97^ These findings have provided new perspectives on the role of the autophagic pathway in driving P2X7R‐mediated pain. Notably, the role of cannabinoid receptor 2 (CB2R) in microglia contrasts with P2X7R; more importantly, its agonist PM226 can reduce pain via inhibiting microglial P2X7R activation.^98^ Therefore, regulating microglial P2X7R is expected to be innovative therapeutic strategy for pain relief.

### 4.2 Microglial P2X7R in inflammatory pain

Inflammatory pain emerges from peripheral tissue injury‐induced inflammatory responses.^56^ Orofacial pain in conditions like pulpitis, periodontitis, caries, or wisdom tooth pericoronitis is a common consequence of oral inflammation.^99^ Microglia in the medullary dorsal horn (MDH) are activated when orofacial pain occurs,^100^, ^101^ and microglial P2X7R is implicated in oral inflammatory diseases and pain transmission. In a model of acute pulpal inflammatory pain induced by mustard oil, the P2X7R antagonists BBG and oxATP significantly alleviated MDH neuron central sensitization, while microglia inhibition using minocycline relieved pain.^102^ In addition, microglial P2X7R plays a pivotal role in dental pulpitis‐induced pain by enhancing NLRP3 and hypoxia‐induced factor‐1α (HIF‐1α) expression.^103^, ^104^ These findings highlight the role of microglial P2X7R in central sensitization and underscore the need for further research into oral inflammatory pain mechanisms.

Microglial P2X7R also involves in other inflammatory diseases. Bmk I, a sodium channel activator and inflammation/pain mediator, was found to upregulate microglial P2X7R expression in the dorsal horn of the spinal cord through plantar injections, activating the p38 pathway and inducing substantial IL‐1β production, ultimately suppressing inflammatory pain.^105^ Similarly, in urological diseases like chronic prostatitis, characterized by painful symptoms, microglial activation in the posterior horn of spinal cord and heightened P2X7R expression were observed.^106^ Overall, P2X7R emerges as a central player in the onset, progression, and regression of multiple inflammation‐induced pain‐related diseases.

### 4.3 The role of microglial P2X7R in cancer‐induced pain

P2X7R has been shown to play a key role in the development of cancer and serve as a target for mediating cancer‐related pain.^107^, ^108^, ^109^, ^110^ Comparative analysis between normal and colorectal cancer samples by Zhang et al.^111^ revealed a significant correlation between elevated P2X7R expression, increased metastasis, and reduced patient survival. Bone cancer pain shares common features of both inflammatory and neuropathic pain, and inhibition of P2X7R in spinal microglia can reduce bone cancer pain by reducing spinal nerve hyperactivity through the p38/IL‐18 pathway.^109^ Furthermore, bone cancer pain can also be relieved by inhibiting NF‐κb/p65, NLRP3‐mediated inflammatory vesicle formation, and IL‐1β expression.^112^ Notably, Huang et al.^31^ demonstrated that P2X7R activation could induce 5‐hydroxytryptamine (5‐HT) release in medullary ventral microglia, which facilitated pain‐sensitive neuron stimulation in the spinal cord, thereby inducing bone cancer pain. Interestingly, Hansen et al.^110^ claimed that heightened bone cancer pain sensitivity in P2X7R knockout mice, underscoring the need to further explore P2X7R's role in this context. While morphine is a frequent analgesic in cancer management, patient tolerance remains a concern. And the spinal microglial P2X7R contributes to morphine tolerance, with intrathecal BBG or P2X7R siRNA intervention sustaining morphine's analgesic efficacy.^113^ Taken together, these findings suggest that P2X7R may be involved in the regulatory mechanisms of cancer pain and imply its potential as a therapeutic target to alleviate cancer pain in patients.

### 4.4 Involvement of P2X7R in other types of pain

In addition to the three most common pain‐related disorders previously discussed, P2X7R has also been implicated in other pain models, such as medication overuse headache, joint pain, and pain–depression comorbidity.^114^, ^115^, ^116^ In a medication overuse headache model, microglial P2X7R has been reported to contribute to NLRP3 inflammasome activation in the trigeminal nucleus caudalis.^117^ Likewise, in rats with monosodium iodoacetate‐induced joint damage, spinal microglial P2X7R was increased due to elevated ATP levels within the cerebral spinal fluid (CSF), impacting pain perception.^91^ Interestingly, in models involving comorbidity of diabetic neuropathic pain and depression, palmatine reduced P2X7R‐GFAP colocalization in the hippocampus, thus mitigating disease symptoms.^118^ These studies suggest that P2X7R in diverse cells is involved in different pain models. In addition, microglial P2X7R plays a role in gastrointestinal disorders, including irritable bowel syndrome, where the P2X7R antagonist GSK1482160 exhibits analgesic efficacy.^119^ Overall, activated glia and P2X7R emerge as key factors in the development of various pain, indicating the potential for interdisciplinary research to position P2X7R as a promising drug target for pain management.

## 5 P2X7R AS A POTENTIAL THERAPEUTIC TARGET FOR PAIN

Due to the widespread presence of P2X7R in the CNS, especially its postsynaptic expression in microglia, P2X7R plays a substantial role in numerous neuroregulatory functions and pain signal transmission. In addition, a series of chronic pain respond to drugs targeting blockade of P2X7R.^107^, ^120^ Therefore, P2X7R as a target has a great prospect for the future development of analgesics.

### 5.1 The function of P2X7R antagonists in preclinical and clinical studies

**FIGURE 3:**
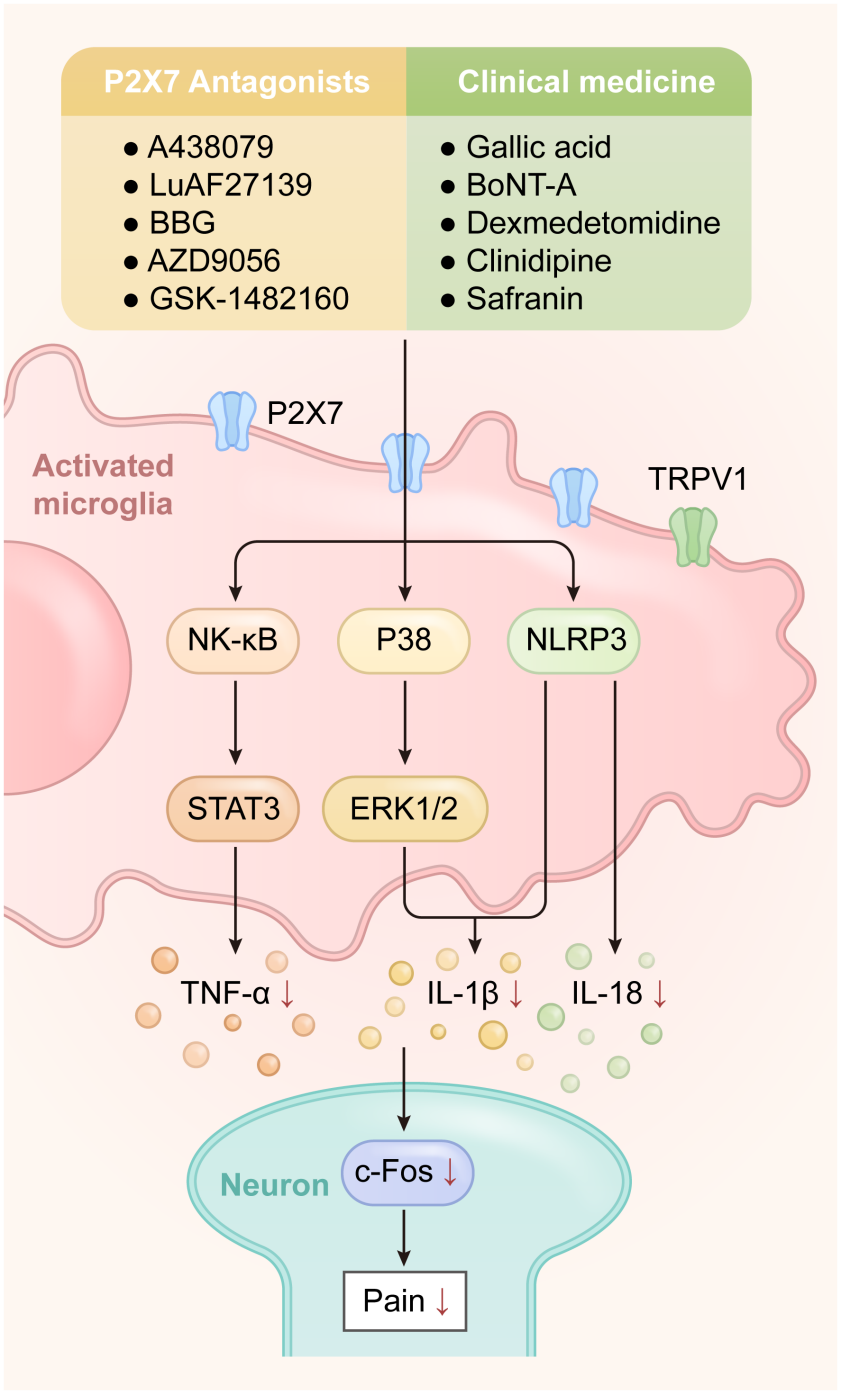
The effect of P2X7R blockade in the development of pain. BBG, A438079, and LuAF27139 are P2X7R antagonists used in preclinical studies to suppress the pain response, while AZD9056 and GSK‐1482160 have entered clinical trials. Similarly, clinical medicine such as gallic acid and safranin also has the ability to relieve pain through P2X7R. These compounds play the analgesic role by inhibiting the NF‐κB/STAT3 and P38/ERK1/2 pathways, reducing the expression of NLRP3 and inflammatory factors including TNF‐α, IL‐1β, and IL‐18, leading to decreased c‐Fos expression and pain relief.

Several P2X7R antagonists have been extensively studied in laboratory investigations involving various pain models, although formal translation to clinical studies is pending. For example, the P2X7R antagonist A438079 effectively reduced the release of inflammatory factors and relieved diabetic neuropathic pain by binding to the P2X7R, modulating the TRPV1 receptor, and subsequently decreasing the phosphorylation levels of p38 and ERK1/2.^43^ In a model of postoperative pain triggered by skin/muscle incision and contraction (SMIR), intrathecal injection of A438079 attenuated SMIR‐induced mechanical nociceptive responses while inhibiting glia activation and TNF‐α upregulation.^121^ Application of A438079 also reduced the expression of P2X7R in salivary gland epithelium, decreased NLRP3‐mediated IL‐1β and IL‐18 expressions, and alleviated inflammation and painful symptoms, as well as salivary gland secretory function.^46^ LuAF27139 was applied in a CCI model to modulate microglia‐derived vesicles release and inhibited the expression of P2X7R.^122^ In addition, the widely used P2X7R antagonist BBG exhibited analgesic effects across various pain models. Moreover, studies have shown that intrathecal BBG administration hindered microglial activation, suppressed c‐Fos expression in the caudal nucleus of the trigeminal nerve, alleviated injury sensations in the trigeminal nervous system, and reversed pain responses in CCI and SMIR models.^121^, ^123^, ^124^ BBG can also alleviate fibromyalgia and bone cancer pain by blocking downstream pathways of P2X7R.^112^, ^125^ Notably, Martins et al.^126^ found that pain responses in cyclophosphamide‐treated animals were eased through P2X7R antagonist pretreatment or P2X7R gene knockdown. Therefore, pharmacological blockade of P2X7R appears to provide an effective strategy for pain relief (Figure 3). On the other hand, several P2X7R antagonists have entered clinical trials for inflammatory conditions and they exert analgesic effects via inhibiting P2X7R‐mediated inflammation responses.^127^ For instance, treatment with AZD9056 can reduce the pain symptom in Crohn's disease patients,^128^ albeit with dose‐dependent gastrointestinal problems.^129^ Despite GSK‐1482160 shows great therapeutic potential in preclinical studies,^130^, ^131^ its insufficient safety margins led to the cessation of further analgesic trials. In addition, several P2X7R antagonist clinical trials failed due to improper design or inadequate validation and negative effects including headache,^132^ back pain, and fatigue.^133^, ^134^ Collectively, the above findings indicate that P2X7R antagonists hold potential for translational and clinical applications in pain management, necessitating continued investigation and development.

### 5.2 The role of clinical medicine related to P2X7R in analgesia

Recent studies have shown that clinically approved specific compounds can be repurposed to pain relief through P2X7R inhibition (Figure 3). Gallic acid, a traditional Chinese medicine, has a strong affinity for P2X7R and can be used to inhibit P2X7R‐mediated NF‐κB and STAT3 pathways, reducing TNF‐α release and suppressing pain in CCI model.^135^ Similarly, botulinum toxin type A (BoNT‐A) can inhibit P2X7R expression and induce the polarization of microglia from M1 to M2 types, thereby increasing the pain threshold of rats.^75^ The sedative dexmedetomidine has been shown to inhibit P2X7R expression and counter injurious spinal microglia effects.^136^, ^137^ Furthermore, the calcium channel blocker cilnidipine can inhibit the activation of the P2X7R/IL‐1β pathway in spinal microglia, thereby ameliorating neuropathic pain.^138^ Safranin has also been shown to inhibit the release of inflammatory factors in facial trigeminal neuralgia models after infraorbital nerve injury, which in turn alleviated pain by decreasing the expression of P2X7R and p38.^139^, ^140^ These findings highlight the potential for re‐purposing existing drugs to expedite effective analgesic compound development.

## 6 CONCLUSION

Advancing novel analgesic drugs remains a pivotal focus in pain research, with recent findings highlighting the significance of P2X7R modulation in pain management. Although it has been clarified that microglial P2X7R plays an important role in pain disorders, the majority have been focused on the peripheral and spinal cord levels; further investigation into the higher brain centers' involvement in pain and related mechanisms is warranted. In addition, it is a major goal for researchers to realize the clinical application of P2X7R antagonists in resolving the pain of patients. To sum up, microglial P2X7R holds promise as a novel pharmacological target for clinical treatments due to its distinctive structure, function, and the application of antagonists. Through multidisciplinary and interdisciplinary researches, an enhanced understanding of microglial P2X7R‐mediated pain pathogenesis will continue to provide new avenues for pain treatment, benefiting patients in need.

## AUTHOR CONTRIBUTIONS

Design Li‐an Wu, Shukai Sun, and Jing Zhang; writing—original draft preparation, Jing Zhang and Lei Gao; writing—review and editing, Jing Zhang, Lei Gao, Yaoyuan Zhang, Haozhen Wang, Shukai Sun, and Li‐an Wu; funding acquisition, Shukai Sun and Li‐an Wu. All authors have read and agreed to the final manuscript.

## FUNDING INFORMATION

This research was funded by grants from the National Natural Science Foundation of China (No. 81771095, 82071235), Key R&D Program of Shaanxi Province (2017SF‐103, 2021KWZ‐26, and 2023‐JC‐ZD‐56) and State Key Laboratory of Military Stomatology (2020ZA01).

## CONFLICT OF INTEREST STATEMENT

The authors declare no conflicts of interest.

## Data Availability

Data sharing is not applicable to this article as no new data were created or analyzed in this study.
